# In Silico Prioritisation of Similarity-Selected Small Molecules Targeting the IsdB NEAT Domain of *Staphylococcus aureus* as a Potential Antivirulence Strategy

**DOI:** 10.3390/ijms27135834

**Published:** 2026-06-28

**Authors:** Warinda Prommachote, Manu Deeudom, Hridek Manimaran, Jittasak Khowsathit, Pimpisid Koonyosying, Bishant Pokharel, Yuvaraj Ravikumar, Somdet Srichairatanakool

**Affiliations:** 1 Department of Biochemistry, Faculty of Medicine, Chiang Mai University, Chiang Mai 50200, Thailand; warinda_p@cmu.ac.th (W.P.); jittasak.khowsathit@cmu.ac.th (J.K.); pimpisid.k@cmu.ac.th (P.K.); bishant_p@cmu.ac.th (B.P.); 2 Department of Microbiology, Faculty of Associated Medical Sciences, Walailak University, Nakhonsrithammarat 80160, Thailand; 3 Division of Bacteriology, Department of Microbiology, Faculty of Medicine, Chiang Mai University, Chiang Mai 50200, Thailand; manu.deeudom@cmu.ac.th; 4 Department of Biotechnology, Acharya Institute of Technology, Soladevanahalli, Karnataka 560107, India; hridekc.22.bebt@acharya.ac.in

**Keywords:** ADMET, antivirulence, Isd, NEAT, molecular docking, molecular dynamics simulation, *Staphylococcus aureus*

## Abstract

The increasing prevalence of multidrug-resistant *Staphylococcus aureus* (MRSA) has necessitated the development of alternative therapeutic strategies targeting bacterial virulence factors. This study employed an integrated in silico approach to identifying potential inhibitors of the iron-regulated surface determinant B Near-iron Transporter domain, a key protein involved in heme acquisition and pathogenicity. Virtual screening and molecular docking identified certain similarity-selected small molecules possessing strong binding affinities, with (4-(1-oxoisoindolin-2-yl)benzoic acid (TOP1) and (4-(2-oxochromen-3-yl)benzoic acid (TOP2) exhibiting the most favorable binding energies at −12.0 and −11.8 kcal/mol, respectively. Molecular dynamics simulations over 200 ns confirmed stable protein–ligand interactions that yielded reduced structural fluctuations in ligand-bound complexes when compared with the apo form. Molecular mechanics/Poisson-Boltzmann surface area (MM/PBSA) analysis revealed that van der Waals interactions were the primary contributors to binding, with TOP1 showing a more favorable overall binding energy. Drug-likeness and pharmacokinetic predictions indicated compliance with Lipinski’s rule of five and moderate bioavailability, although limited intestinal absorption was observed. Toxicity predictions indicated that both compounds are non-mutagenic but may exhibit hepatotoxicity. Notably, TOP1 exhibited potential nephrotoxicity, cardiotoxicity, and carcinogenicity, whereas TOP2 demonstrated a more favorable safety profile. These findings highlight a trade-off between binding affinity and safety, suggesting that TOP2 emerged as a computationally prioritized candidate for future experimental validation. Because the present findings represent computational predictions only, further orthogonal computational analyses and experimental studies are required to confirm the proposed binding modes, biological activity, and therapeutic potential of the identified compounds.

## 1. Introduction

*Staphylococcus aureus* is a ubiquitous opportunistic pathogen that is responsible for a wide spectrum of infections, ranging from superficial skin and soft tissue infections to severe systemic diseases such as bacteremia, endocarditis, and various implant-associated infections [1,2,3]. The global burden of *S. aureus* infections remains substantial, with increasing morbidity and mortality rates being reported worldwide [4,5]. Its ability to form biofilms further complicates treatment, particularly in implanted medical devices such as prosthetic joints and cardiovascular implants, which can often lead to persistent infections and treatment failure [6,7]. Of particular concern is the emergence and spread of MRSA, which has significantly reduced the effectiveness of conventional antibiotics and has further complicated clinical management [8,9,10]. This growing challenge underscores the urgent need for alternative therapeutic strategies that complement existing antimicrobial approaches.

Iron acquisition is a critical determinant of bacterial survival and virulence as an infection emerges, as host defense mechanisms restrict iron availability through nutritional immunity [11,12]. To overcome this limitation, *S. aureus* has evolved specialized iron uptake systems that include siderophore-mediated transport and heme acquisition pathways [13,14,15]. Among these, the iron-regulated surface determinant B (IsdB) Near-iron Transporter (NEAT) domain system plays a central role in heme capture and transport from host hemoproteins [11,16,17]. The IsdB protein, a key hemoglobin receptor containing NEAT domains, is essential for bacterial fitness under iron-limited conditions [18,19]. Because NEAT domains are surface-exposed and directly involved in virulence rather than viability, they represent attractive targets for antivirulence strategies that may reduce selective pressure for resistance development [16].

Structure-based computational approaches are increasingly being used to prioritize antimicrobial targets and lead compounds, particularly during early-stage discovery where experimental screening is resource-intensive [20,21]. In silico methods, such as molecular docking and molecular dynamics simulations (MDS), enable the systematic evaluation of ligand–target interactions and provide mechanistic insight into binding stability and interaction profiles [21,22]. Within the context of antimicrobial resistance research, these approaches are valuable for identifying antivirulence candidates that may complement antibiotic susceptibility testing and resistance management strategies [23].

In clinical settings, the rising prevalence of MRSA has further diminished the efficacy of conventional antibiotics and reinforced the need for alternative therapeutic concepts. Although the present study does not evaluate antimicrobial resistance phenotypes or compound their efficacy in resistant strains, genome-based and computational analyses provide a robust framework for identifying conserved virulence-associated targets. Such targets may inform the development of antivirulence strategies that are intended to complement, rather than replace, existing antimicrobial therapies. Accordingly, this study applies an integrated in silico workflow that combines virtual screening, molecular docking, MDS, and binding free-energy calculations to prioritize the similarity-selected small molecules that target the IsdB NEAT domain of *S. aureus*, thereby establishing computationally informed candidates to support future experimental validation.

## 2. Results

### 2.1. Docking Validation Results

Prior to virtual screening, the docking protocol was validated through a redocking procedure that used the co-crystallized heme ligand. The predicted binding poses closely matched the experimentally determined conformation, yielding a root mean square deviation (RMSD) value of ≤0.02 Å, which is unusually low and reflects near perfect reproduction of the crystallographic pose. This value falls within the acceptable threshold (≤2.0 Å), indicating that the docking protocol is reliable for reproducing ligand binding modes within the IsdB NEAT domain. Consequently, the validated docking parameters were applied to screen the similarity-selected small molecules.

### 2.2. Virtual Screening and Molecular Docking Analysis

**Figure 1:**
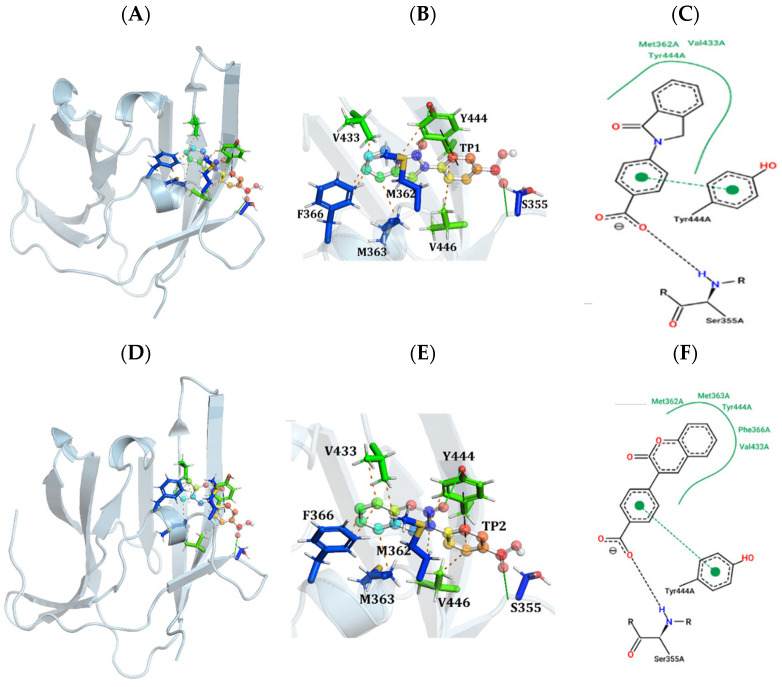
Molecular docking analysis of similarity-selected small molecules with the IsdB NEAT domain (PDB ID: 3RTL). (**A**) Illustration of the IsdB NEAT domain complexed with TOP1. (**B**) Three-dimensional stick representation of the binding pocket illustrating interactions between TOP1 and key residues. (**C**) Two-dimensional interaction diagram showing hydrogen bonding and hydrophobic contacts between TOP1 and the active-site residues. (**D**) Cartoon representation of the IsdB NEAT domain complexed with TOP2. (**E**) Three-dimensional stick representation of the binding pocket illustrating interactions between TOP2 and key residues. (**F**) Two-dimensional interaction diagram showing hydrogen bonding and hydrophobic contacts between TOP2 and the active-site residues. In the (**A**,**B**,**D**,**E**), the interacting amino acids are represented as sticks as shown in blue and green color, while the ligands is show in rainbow color and represented as ball and stick model.

Molecular docking analysis of the screened compounds against the IsdB NEAT domain (PDB ID: 3RTL) revealed strong binding affinities among the top-ranked candidates, with Vina scores ranging from −10.4 to −12.0 kcal/mol. Among these, TOP1 exhibited the most favorable binding energy (−12.0 kcal/mol), followed closely by TOP2 (−11.8 kcal/mol), indicating high binding stability within the heme-binding pocket. As shown in Figure 1, both compounds occupy a similar binding cavity and establish extensive interactions with key residues, including M362, M363, F366, V433, and Y444, which were consistently identified across the top-ranked ligands (Table 1).

Detailed interaction analysis demonstrated that TOP1 forms a single hydrogen bond with S355, complemented by multiple hydrophobic and π-interactions with surrounding residues, thereby contributing to its superior binding affinity. In contrast, TOP2 forms two hydrogen bonds with S355 and Y444, as visualized in the two-dimensional interaction diagrams, but exhibits slightly reduced binding affinity when compared with TOP1. This suggests that while hydrogen bonding contributes to ligand stabilization, non-covalent interactions, particularly hydrophobic contacts within the binding pocket, play a dominant role in determining the overall binding strength. Collectively, these findings highlight the importance of the conserved hydrophobic core of the IsdB NEAT domain in ligand recognition and support the prioritization of TOP1 and TOP2 as promising candidates for further investigation. In addition to the top-ranked compounds, docking poses and interaction profiles for the remaining screened ligands (TOP3–TOP10) are provided in the Supplementary Table S1 and Figures S1 and S2, thereby illustrating consistent binding within the IsdB NEAT domain active site.

### 2.3. Molecular Dynamics Analysis of Protein-Ligand Stability

**Figure 2:**
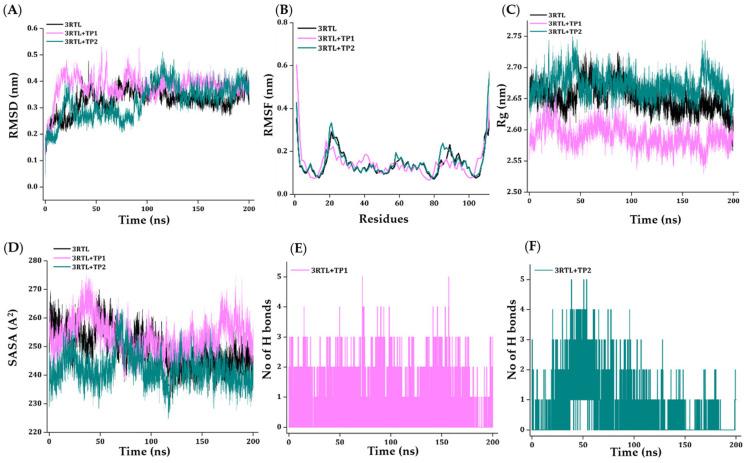
Molecular dynamics simulation analysis of 3RTL and ligand-bound complexes (3RTL–TP1 and 3RTL–TP2) over a 200 ns simulation period. (**A**) Root mean square deviation (RMSD) of the protein backbone against the simulation time, depicting the overall structural stability of the apo protein (3RTL) and ligand-bound complexes (3RTL–TP1 and 3RTL–TP2) throughout the simulation. (**B**) Root mean square fluctuation (RMSF) per residue determined from the MD trajectories, illustrating the flexibility of individual amino acid residues in the apo and ligand-bound systems (3RTL–TP1 and 3RTL–TP2). Peaks indicate regions exhibiting higher conformational mobility. (**C**) Radius of gyration (Rg) profiles of the apo protein and ligand-bound complexes (3RTL–TP1 and 3RTL–TP2) during the 200 ns simulation, representing changes in protein compactness and overall structural packing upon ligand binding. (**D**) Solvent-accessible surface area (SASA) as a function of simulation time for the apo protein and ligand-bound systems (3RTL–TP1 and 3RTL–TP2), providing insight into changes in protein surface exposure to the solvent environment. (**E**) Number of intermolecular hydrogen bonds formed between TP1 and 3RTL during the simulation, demonstrating the stability and consistent existence of ligand–protein interactions over time. (**F**) Number of intermolecular hydrogen bonds formed between TP2 and 3RTL during the simulation, demonstrating the stability and consistent existence of ligand–protein interactions over time. Color Indications: Apo (3RTL), black; 3RTL–TP1 complex, magenta; and 3RTL–TP2 complex, cyan.

To further evaluate the stability and dynamic behavior of the protein–ligand complexes, MDS were performed for the apo IsdB NEAT domain and the TOP1–3RTL and TOP2–3RTL complexes over 200 ns (Figure 2). The RMSD profiles (Figure 2A) indicated that both ligand-bound systems reached equilibrium with relatively stable trajectories when compared with the apo structure, suggesting enhanced structural stability upon ligand binding. Residue-level flexibility analysis using root mean square fluctuation (RMSF) (Figure 2B) showed reduced fluctuations in key binding-site regions for the ligand-bound complexes, particularly for the TOP1–3RTL system. The radius of gyration (Rg) profiles (Figure 2C) demonstrated consistent structural compactness throughout the simulation, with minimal deviation observed for both complexes. Similarly, solvent-accessible surface area (SASA) analysis (Figure 2D) indicated stable surface exposure, reflecting the maintenance of protein structural integrity. Hydrogen bond analysis revealed that both ligands formed stable intermolecular interactions with the protein over time, with TOP2 exhibiting a slightly higher number of hydrogen bonds when compared with TOP1 (Figure 2E,F).

Despite this, the overall stability and reduced flexibility observed for the TOP1 complex suggest a more favorable binding profile, consistent with the docking results.

### 2.4. Binding Free-Energy Estimation by MM/PBSA

**Figure 3:**
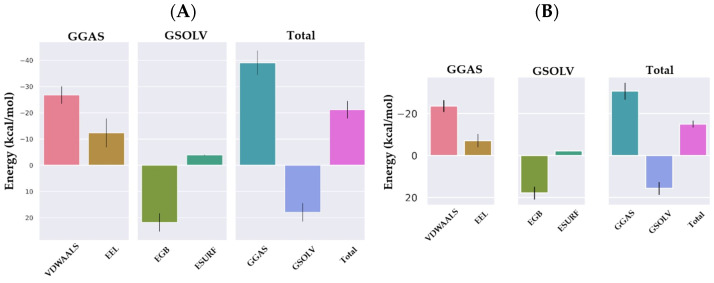
Binding free-energy decomposition of IsdB NEAT domain (3RTL) complexes with similarity-selected small molecules calculated using the MM/PBSA method. (**A**) Energy components contributing to the binding free energy of the 3RTL–TOP1 complex. (**B**) Energy components contributing to the binding free energy of the 3RTL–TOP2 complex. The total binding free energy is decomposed into van der Waals, electrostatic, polar solvation, and nonpolar solvation contributions, highlighting the dominant interactions governing ligand binding. Data are expressed as mean ± SD values.

To quantitatively estimate the binding affinities and energetic contributions governing ligand binding, MM/PBSA calculations were performed on the MDS trajectories of the TOP1–3RTL and TOP2–3RTL complexes (Figure 3). The binding free-energy decomposition revealed that van der Waals and electrostatic interactions contributed favorably to ligand binding in both systems, whereas polar solvation energy exhibited an unfavorable contribution, partially offsetting the total binding energy. Notably, the TOP1 complex demonstrated more favorable capacity for the overall binding of free energy when compared with TOP2, which was consistent with the docking results and molecular docking stability profiles. Further analysis indicated that van der Waals interactions were the dominant driving force for binding in both complexes, highlighting the importance of hydrophobic interactions within the heme-binding pocket. Although TOP2 exhibited relatively strong electrostatic contributions, these were counterbalanced by higher polar solvation penalties. In contrast, the TOP1 complex showed a more balanced energy profile, resulting in a more stable and energetically favorable interaction with the IsdB NEAT domain. These findings reinforce the critical role of nonpolar interactions in ligand stabilization and support the prioritization of TOP1 as the most promising candidate among the screened compounds.

### 2.5. In Silico Drug-Likeness, Pharmacokinetics, and Toxicity

To evaluate the drug-likeness and pharmacokinetic properties of the top-ranked compounds, the physicochemical parameters of TOP1 and TOP2 were predicted using the SwissADME web server (Table 2). Both compounds exhibited molecular weights below 500 g/mol and favorable lipophilicity values (logP < 5), which were consistent with Lipinski’s rule of five. Additionally, both compounds were predicted to be highly soluble, which may support adequate bioavailability. The topological polar surface area (TPSA) values for TOP1 and TOP2 were within an acceptable range for oral bioavailability, although slightly elevated, suggesting potential limitations in membrane permeability. Notably, TOP1 displayed no rotatable bonds, indicating a more rigid structure, whereas TOP2 exhibited moderate flexibility with four rotatable bonds. The fraction of sp^3^-hybridized carbon atoms (fraction cSP^3^) was higher in TOP2, suggesting greater three-dimensional characteristics; however, the corresponding value for TOP1 should be interpreted with caution until verified from the original SwissADME output. Both compounds showed comparable bioavailability scores (0.56), indicating moderate oral bioavailability potential. Overall, these results suggest that TOP1 and TOP2 possess favorable drug-like properties and physicochemical characteristics that are suitable for further optimization. However, the reported fraction cSP^3^ value for TOP1 requires verification, as negative values are chemically implausible and may reflect a reporting or calculation error.

To further assess the pharmacokinetic and toxicity profiles of the selected compounds, as well as the relevant absorption, distribution, metabolism, excretion, and toxicity (ADMET) properties, of TOP1 and TOP2 were predicted using pharmacokinetics via graph-based signature (pkCSM) and ProTox-II web servers (Table 3). Both compounds exhibited moderate water solubility and relatively low permeability (logPS) values for the human colon adenocarcinoma cell line (Caco-2), suggesting limited passive diffusion across intestinal epithelial cells. Consistent with these findings, both compounds were predicted to have low intestinal absorption values (<30%). Distribution analysis revealed moderate plasma protein binding, as reflected by fraction unbound values of 0.15 and 0.10 for TOP1 and TOP2, respectively. Regarding blood–brain barrier (BBB) penetration (logBBB) values, TOP1 exhibited borderline BBB permeability (logBBB = 0.011), whereas TOP2 showed poor BBB permeability (logBBB = −1.383), suggesting limited central nervous system exposure, particularly with regard to TOP2. Similarly, negative CNS permeability (logPS) values for both compounds further support restricted brain penetration. Metabolically, TOP1 was not predicted to be a substrate of cytochrome (CYP) 3A4 and showed inhibitory activity toward CYP1A2 or CYP2C19, whereas TOP2 was predicted to be both a CYP3A4 substrate and a CYP1A2 inhibitor. However, both compounds were predicted to inhibit CYP2C9 but not CYP2D6, indicating the potential for specific metabolic drug–drug interactions. Excretion analysis showed moderate predicted total clearance values for both compounds. Toxicity predictions indicated that TOP1 and TOP2 are non-mutagenic based on Ames test results and exhibited relatively low acute toxicity based on the median lethal dose (LD_50_) values. However, neither compound was predicted to exhibit hepatotoxic capabilities according to the ProTox-II analysis, which may represent a potential limitation that would require further experimental validation. Collectively, these findings suggest that while both compounds possess generally acceptable pharmacokinetic and safety profiles, their limited intestinal absorption and permeability may require further optimization to improve the oral bioavailability and relevant drug-like properties.

Both TOP1 and TOP2 were predicted to be inactive across multiple nuclear receptor and stress response pathways, including peroxisome proliferator-activated activator gamma (PPAR-γ), nuclear factor erythroid 2-related factor 2 (Nrf2)/antioxidant responsive element (ARE), heat-shock factor response, mitochondrial membrane potential (MMP), p53 signaling, and ATPase family AAA domain containing 5 (ATAD5). A more comprehensive toxicity evaluation using ProTox-II (Table 4) revealed notable differences between the two compounds. This indicates a low likelihood of endocrine disruption, oxidative stress induction, mitochondrial dysfunction, or genotoxic effects. Despite these favorable pathway-level safety profiles, TOP1 exhibited predicted organ-specific toxicities, suggesting that its adverse effects may arise from off-target interactions rather than global cellular stress mechanisms. In contrast, TOP2 demonstrated both pathway-level safety and absence of organ toxicity, supporting its prioritization as a safer lead compound.

## 3. Discussion

The present study employed an integrated in silico strategy to identify and characterize similarity-selected small molecules that target the IsdB NEAT domain of *S. aureus*, a key virulence factor involved in heme acquisition. Targeting virulence-associated pathways rather than bacterial viability has emerged as a promising strategy to mitigate antimicrobial resistance by reducing selective pressure for resistance development [24,25]. Molecular docking analysis identified TOP1 and TOP2 as the most promising candidates, exhibiting strong binding affinities (−12.0 and −11.8 kcal/mol, respectively). Both compounds interacted with conserved residues within the heme-binding pocket, including M362, M363, F366, V433, and Y444, which are critical for ligand stabilization and heme coordination. A key finding of this study is the consistent involvement of these hydrophobic residues across all top-ranked ligands, highlighting the central role of the conserved binding pocket in ligand recognition and suggesting that it represents a structurally robust target for inhibitor design.

IsdB is a critical hemoglobin receptor that enables *S. aureus* to acquire heme-derived iron and is essential for heme acquisition under iron-restricted host conditions imposed by host nutritional immunity [26,27,28]. Accordingly, the inhibition of IsdB may be impaired iron uptake and may influence bacterial fitness without directly affecting bacterial viability, thereby potentially reducing selective pressure for resistance development. Previous studies have demonstrated that IsdB contributes significantly to bacterial fitness, iron acquisition, and virulence during infection [17,28]. Consequently, inhibition of IsdB-mediated heme uptake may impair bacterial growth and pathogenicity without directly affecting bacterial viability. This antivirulence strategy is particularly attractive because it may impose lower selective pressure for the emergence of antimicrobial resistance when compared with conventional bactericidal or bacteriostatic antibiotics. Herein, although TOP2 formed more hydrogen bonds, TOP1 demonstrated superior binding affinity, indicating that hydrophobic and van der Waals interactions play a dominant role in ligand stabilization within the IsdB NEAT domain. This finding reinforces the conclusions drawn from previous reports, which found that nonpolar interactions often outweigh hydrogen bonding in determining binding strength within hydrophobic protein pockets [29,30]. Importantly, this study provides new insight into the interaction landscape of the IsdB NEAT domain by quantitatively linking docking results with dynamic stability and energetic contributions. Therefore, compounds capable of disrupting IsdB function could serve as valuable adjunctive agents for the management of *S. aureus* infections, including those caused by MRSA. Furthermore, MDS supported the stability of the protein–ligand complexes. The study employs a small 200 ns simulation time. Albeit the simulation time used in this study does not was long but our observation during this 200 ns time showed that all the systems reached stability and hence were considered. Both TOP1 and TOP2 maintained stable RMSD profiles and exhibited reduced residue-level fluctuations when compared with the apo structure, suggesting enhanced structural rigidity upon ligand binding. Notably, the TOP1 complex showed lower flexibility in key binding-site regions, indicating a more stable interaction pattern. These results are consistent with previous computational studies demonstrating that ligand binding can stabilize protein conformations and reduce dynamic fluctuations [31]. The results implied here although demonstrate that the calculated fluctuations and associated deviations observed in the RMSD, RMSF, Rg and SASA are relatively small and fall within, or below, the range commonly reported in comparable molecular dynamics studies, the inclusion of direct comparison along with statistical validation is of utmost importance and will further warrant the study findings.

Binding free-energy calculations using MM/PBSA revealed that van der Waals interactions are the primary driving force for binding, while polar solvation energy can contribute unfavorably. This energetic profile is characteristic of hydrophobic binding pockets and further supports the dominant role of nonpolar interactions in stabilizing ligand binding [32]. The more favorable binding free energy observed for TOP1 corroborates the molecular docking findings, reinforcing its potential as a high-affinity inhibitor. Drug-likeness evaluation indicated that both compounds comply with Lipinski’s rule of five and exhibit favorable physicochemical properties. However, ADMET predictions revealed moderate intestinal absorption and low permeability capabilities, suggesting potential limitations in oral bioavailability. These findings highlight a common challenge in drug design, where increased polarity can improve solubility but reduce membrane permeability [33].

An important observation of this study is the apparent trade-off between the predicted binding affinity and the predicted toxicity. While TOP1 demonstrated superior binding affinity and stability, toxicity predictions indicated potential nephrotoxicity, cardiotoxicity, and carcinogenicity. In contrast, TOP2 exhibited a more favorable safety profile by being inactive for these toxicity endpoints. Both compounds were predicted to exhibit hepatotoxicity, which remains a common concern in drug development and underscores the importance of early toxicity screening [1]. These results suggest that TOP2 may represent a more viable lead compound despite slightly lower binding affinity, highlighting the importance of balancing efficacy and safety in line with lead optimization.

Despite these promising findings, several limitations should be acknowledged. First, all results are based on computational predictions, which may not fully capture the complexity of biological systems. Molecular docking and MDS rely on force fields and approximations that may introduce uncertainties in binding affinity estimation. Second, the MM/PBSA method, although widely used, provides approximate free-energy calculations and may not fully account for entropic contributions. Alternative docking algorithms and receptor-flexibility approaches may generate different binding conformations and were not evaluated in the present study. Importantly, our findings represent computational predictions only and should therefore be interpreted as hypothesis-generating results rather than definitive evidence of inhibitory activity. The molecular docking, molecular dynamics simulation, and MM/PBSA workflows employed in this study involve methodological choices and parameter selections that may influence ligand ranking, predicted binding modes, and estimated binding energies. Alternative docking algorithms, receptor-flexibility treatments, side-chain rotamer sampling approaches, scoring functions, and free-energy estimation methods may generate different outcomes and were not evaluated in the present work. Consequently, additional benchmarking using orthogonal computational strategies would further strengthen confidence in the predicted binding modes and ligand prioritization. Furthermore, molecular dynamics simulations were conducted for 200 ns, which was considered sufficient to evaluate the overall stability of the selected complexes within the scope of this study. Although raw trajectories were retained to preserve the underlying dynamic behavior and avoid masking transient fluctuations, moving-average analyses and mean ± SD values calculated from the final 50 ns were also examined to facilitate the interpretation of long-term stability trends. Nevertheless, additional statistical analyses of the trajectories may provide further insight into the dynamic behavior of the complexes. Similarly, although the MM/PBSA standard deviations observed in this study were lower than those commonly reported in some computational investigations, the calculations were performed using a consistent trajectory-sampling procedure and should be interpreted within the limitations of the selected methodology. Future studies employing consensus docking, induced-fit docking, MM/GBSA, free-energy perturbation methods, and experimental validation will be valuable for confirming the robustness and biological relevance of the identified lead compounds. Additionally, ADMET and toxicity predictions were derived from in silico models and should be interpreted with caution, as they require experimental validation. The predicted hepatotoxicity and other toxicity endpoints, particularly for TOP1, may not directly translate to in vivo outcomes. Another limitation is the lack of evaluation of compound activity against different strains of *S. aureus*, including clinically relevant resistant isolates such as MRSA. Furthermore, this study did not investigate ligand selectivity or potential off-target interactions, which are critical factors in drug development. The reported fraction cSP^3^ value for TOP1 would also require verification to ensure data accuracy prior to the experimental progression.

Future work should focus on experimental validation of the identified compounds through in vitro assays to confirm their inhibitory activity against the IsdB NEAT domain and their impact on bacterial virulence. Structural optimization of the lead compounds is recommended to improve pharmacokinetic properties, particularly intestinal absorption and membrane permeability, while minimizing toxicity. Advanced computational approaches, such as free-energy perturbation calculations, may provide more accurate binding affinity estimates and guide rational drug design. Additionally, selectivity studies should be conducted to assess potential off-target effects and improve specificity toward the IsdB NEAT domain. In vivo studies are essential to evaluate the pharmacokinetics, toxicity, and therapeutic efficacy of the compounds in relevant infection models. Investigating the activity of these compounds against multidrug-resistant strains, including MRSA, will further establish their potential as antivirulence agents. Finally, a combination of studies with existing antibiotics may provide insight into any relevant synergistic effects and support the development of combination therapies to combat antimicrobial resistance.

## 4. Materials and Methods

### 4.1. Overall Computational Workflow

This study employed an integrated structure-based computational workflow to identify and prioritize the screened compounds targeting the IsdB NEAT domain of *S. aureus*. The workflow comprised ligand library preparation, molecular docking with validation, MDS, binding free-energy calculations, and in silico pharmacokinetic and toxicity predictions.

### 4.2. Ligand Preparation and Virtual Screening

#### 4.2.1. Ligand Library Construction

A compound library was generated from the PubChem database, which contains over 96 million chemical structures [34]. A similarity-based search was performed in the PubChem database using an oxazole-containing reference compound as the query structure. Compounds exhibiting ≥90% structural similarity based on the PubChem fingerprint/Tanimoto coefficient were retrieved. Because similarity searching is based on overall molecular fingerprints rather than strict scaffold preservation, the resulting library included compounds that shared related structural features with the query molecule but did not necessarily retain the oxazole ring system.

#### 4.2.2. Ligand Preparation

The selected compounds were downloaded in Structure Data File format. Ligands were prepared by assigning proper bond orders, adding hydrogen atoms, and performing energy minimization. Structural visualizations and inspections were carried out using PyMOL version 3.1 (Schrödinger, Inc., New York, NY, USA) [35].

### 4.3. Protein Preparation

The crystal structure of the IsdB NEAT domain from *S. aureus* (PDB ID: 3RTL) was retrieved from the Research Collaboratory for Structural Bioinformatics Protein Data Bank (RCSB PDB) [36]. Protein preparation was performed using BIOVIA Discovery Studio Visualizer v21.1.020298 (Dassault Systèmes, Waltham, MA, USA). Water molecules were removed, hydrogen atoms were added, bond orders were assigned, and missing residues were corrected where necessary. Protonation states of ionizable residues were adjusted to maintain appropriate hydrogen-bonding interactions under physiological conditions.

### 4.4. Docking Protocol Validation

#### 4.4.1. Redocking Procedure

To validate the docking protocol, a redocking procedure was performed using the co-crystallized heme ligand extracted from the 3RTL structure. The ligand was removed from the binding site and prepared by adding hydrogen atoms and assigning appropriate bond orders. The prepared ligand was re-docked into the original binding site using AutoDock Vina v1.2.5 [37], employing the same grid box dimensions, center coordinates, and docking parameters used for subsequent virtual screening.

#### 4.4.2. RMSD Calculation and Validation Criteria

The accuracy of the docking protocol was evaluated by comparing the predicted ligand pose with the experimentally determined crystallographic conformation. Structural alignment was performed using PyMOL Molecular Graphics System v3.0 (Schrödinger, LLC, New York, NY, USA) [35], and the RMSD value between the heavy atoms of the docked and crystal ligand was calculated. An RMSD value ≤ 2.0 Å was considered indicative of a reliable docking protocol [21,22]. This validation ensured that the docking setup could accurately reproduce native ligand binding modes prior to screening.

### 4.5. Molecular Docking

#### 4.5.1. Docking Setup

Molecular docking was carried out using AutoDock Vina v1.2.5 [38]. The docking grid box was centered on the heme-binding pocket of the IsdB NEAT domain, based on the coordinates of the co-crystallized ligand.

#### 4.5.2. Docking Execution and Ranking

All ligands were docked using identical parameters to ensure consistency. Docking poses were ranked according to predicted binding affinities (kcal/mol), and the top ten compounds were selected for further analysis.

#### 4.5.3. Interaction Analysis

Protein–ligand interactions, including hydrogen bonding and hydrophobic contacts, were analyzed using BIOVIA Discovery Studio Visualizer v21.1.0.20298 (Dassault Systèmes BIOVIA, San Diego, CA, USA) and the PyMOL [35].

### 4.6. Molecular Dynamics Simulations

MDS were performed for the apo IsdB NEAT domain and the TOP1–3RTL and TOP2–3RTL complexes over 200 ns. Each system was prepared by assigning the appropriate protein and ligand force-field parameters, followed by solvation in an explicit water box and neutralization with counterions. Energy minimization was conducted to remove unfavorable contacts, followed by equilibration under constant volume and constant pressure conditions. Production simulations were subsequently performed under appropriate physiological temperature and pressure conditions. Trajectory coordinates were saved at regular intervals and analyzed for root mean square deviation (RMSD), root mean square fluctuation (RMSF), radius of gyration (Rg), solvent-accessible surface area (SASA), and intermolecular hydrogen-bond formation.

#### 4.6.1. System Setup

The top two protein–ligand complexes (TOP1–3RTL and TOP2–3RTL), along with the apo protein, were subjected to molecular dynamics simulations using GROningen Machine for Chemical Simulation (GROMACS) version 2019.4 [38] with the CHARMM36 force field. Each system was solvated in a cubic box using the TIP3P water model and neutralized with Na^+^ and Cl^−^ ions.

#### 4.6.2. Energy Minimization and Equilibration

Energy minimization was performed using the steepest descent algorithm. Systems were equilibrated under constant normal volume, temperature, and pressure.

#### 4.6.3. Production Simulation

Production of MDS was carried out for 200 ns at 310 K.

#### 4.6.4. Trajectory Analysis

Trajectory analyses were performed to evaluate structural stability and dynamics: RMSD, RMSF, Rg, SASA, and hydrogen bond analysis. These analyses provided insight into protein stability, flexibility, and ligand interaction persistence.

### 4.7. Binding Free-Energy Calculations

#### 4.7.1. MM/PBSA Analysis

Binding free energies were calculated using the MM/PBSA method implemented via the g_mmpbsa tool [39]. MM/PBSA calculation was performed using gmx_MM/PBSA. A total of 1000 snapshots extracted from the final 50 ns of the molecular docking trajectories at 50 ps interval were analyzed. Binding free energies of the TOP1–3RTL and TOP2–3RTL complexes were estimated using the MM/PBSA method. Calculations were performed using trajectory snapshots extracted from the equilibrated portion of the molecular dynamic simulations. The total binding free energy was decomposed into van der Waals, electrostatic, polar solvation, and nonpolar solvation energy contributions. Binding-energy values are presented as mean ± standard deviation values and were calculated from the analyzed trajectory frames.

#### 4.7.2. Free Energy Calculation

Binding free energy (ΔG_binding_) was calculated using Equation (1) as follows:

ΔG_binding_ = G_complex_ − (G_protein_ + G_ligand_) (1)

where G_complex_, G_protein_, and G_ligand_ represent the free energies of the complex, protein, and ligand, respectively.

#### 4.7.3. Energy Decomposition

Energy contributions included van der Waals interactions, electrostatic interactions, polar solvation energy, and nonpolar solvation energy. These components were analyzed to determine the dominant forces governing ligand binding.

### 4.8. Drug-Likeness and ADMET Prediction

#### 4.8.1. Physicochemical and Drug-Likeness Analysis

Drug-likeness properties were evaluated using the SwissADME web server (http://www.swissadme.ch/ accessed on 10 February 2026) [40]. The parameters assessed included molecular weight, logP, hydrogen bond donors and acceptors, and Lipinski’s rule of five compliance.

#### 4.8.2. Pharmacokinetic Predictions

Pharmacokinetic properties, including ADMET, were predicted using the pkCSM web server [40].

#### 4.8.3. Toxicity Assessment

Toxicity profiles were evaluated using the ProTox-II server (Structural Bioinformatics Group, Institute for Physiology & Experimental and Clinical Research Center, Charité, University Medicine, Philippstrasse, Berlin associated with Freie Universität Berlin, Germany) [41], which was employed to predict acute toxicity and any potential adverse effects, thereby supporting early-stage prioritization of the candidate compounds.

## 5. Conclusions

This study demonstrates the effectiveness of an integrated computational approach for identifying potential antivirulence agents targeting the IsdB NEAT domain of *Staphylococcus aureus*. Molecular docking, molecular dynamics simulations, and MM/PBSA analyses consistently identified TOP1 and TOP2 as high-affinity ligands, with TOP1 exhibiting superior binding strength driven primarily by hydrophobic interactions. However, ADMET and toxicity predictions revealed a critical balance between efficacy and safety. While TOP1 showed stronger binding affinity, its predicted toxicity profile may limit its therapeutic potential. In contrast, TOP2 demonstrated a more favorable safety profile with acceptable pharmacokinetic properties, making it a more suitable candidate for further development. These findings support the feasibility of targeting the IsdB NEAT domain as an antivirulence strategy and highlight similarity-selected small molecules as promising scaffolds for drug development. Nevertheless, the present findings represent computational predictions only and should be regarded as a framework for future hypothesis-driven investigations rather than conclusive evidence of biological efficacy. Future work should focus on experimental validation, structural optimization, and the evaluation of biological activity to advance these compounds toward clinical applications.

## Figures and Tables

**Table 1:** Docking results against the IsdB NEAT domain (PDB ID: 3RTL).

Ligand	PubChem ID	Hydrogen Bonds	H-Bonding Residues	Non-Covalent Interactions	Non-Covalent Interaction Residues	Vina Score (kcal/mol)
TOP1	13190987	1	S355	6	F366, V433, Y444, M362, M363, V446	−12.0
TOP2	675423	2	S355, Y444	5	V446, M362, M363, F366, V433	−11.8
TOP3	53228598	1	S361	6	M362, M363, F366, Y440, V443, Y444	−11.5
TOP4	684763	0	-	7	Y391, F366, M363, V446, Y444, G442, V433, H434	−11.3
TOP5	721996	1	M363	8	F366, Y391, V435, V433, Y440, V431, M362, V446	−11.2
TOP6	832171	0	-	6	Y391, W392, V433, V431, Y444, M362, M363	−10.8
TOP7	872756	1	M363	4	F366, V433, M362, M363	−10.8
TOP8	889081	1	Y440	7	Y391, V435, V433, V446, M362, M363, F366	−10.7
TOP9	890848	1	M363	3	F366, V433, Y444	−10.5
TOP10	976591	0	-	5	Y391, Y440, Y444, V433, M362, M363	−10.4

**Table 2:** Predicted physicochemical properties and drug-likeness parameters of selected similarity-selected small molecules (TOP1 and TOP2) evaluated using the SwissADME web server. Parameters include molecular weight, logP, TPSA, hydrogen bond donors and acceptors, solubility, fraction cSP^3^, number of rotatable bonds, and bioavailability score, all of which were assessed according to Lipinski’s rule of five.

Parameter	TOP1	TOP2
Lipophilicity (logP)	2.7	3.08
MW (g/mol)	253.25	266.25
Solubility	Very soluble	Very soluble
TPSA (A2)	75	68.37
Rotatable bonds	2	2
Fraction cSP^3^	0.12	0.22
Bioavailability score	0.55	0.55
Bioradar plot	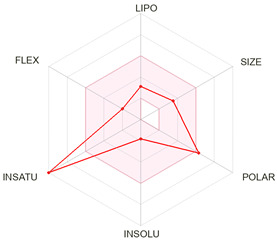	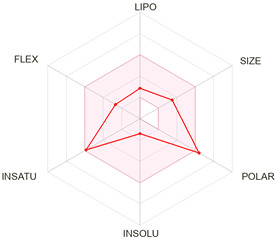

Fraction cSP^3^ for TOP1 could not be confirmed from the original SwissADME output and was therefore not interpreted.

**Table 3:** Predicted ADMET properties of TOP1 and TOP2 compounds evaluated using pkCSM and ProTox-II web servers. Parameters include water solubility, intestinal absorption, Caco-2 permeability, BBB permeability, CYP450 interactions, total clearance, and toxicity endpoints such as Ames mutagenicity, LD_50_, and hepatotoxicity.

Domain	Parameter	TOP1	TOP2
Absorption	Water solubility (log mol/L)	−2.89	−2.856
	Caco-2 permeability (log P_app_, 10^−6^ cm/s)	−4.574	−0.685
	Intestinal absorption (%)	24.7%	28.9%
Distribution	Fraction unbound (human)	0.15	0.10
	BBB permeability (logBBB)	0.011	−1.385
	CNS permeability (logPS)	−1.5	−1.2
Metabolism	CYP2D6 substrate	No	No
	CYP3A4 substrate	No	Yes
	CYP1A2 inhibitor	No	Yes
	CYP2C19 inhibitor	No	No
	CYP2C9 inhibitor	Yes	Yes
Excretion	Total clearance (log mL/min/kg)	0.621	0.471
	Renal OCT2 substrate	1.38	1.203
Toxicity	Ames mutagenicity	No	No
	Maximum tolerated dose (human; log mg/kg/day)	0.5	0.5
	Oral rat acute toxicity LD_50_ (mol/kg)	2.0	2.5
	Predicted hepatotoxicity	No	No

BBB = blood–brain barrier, Caco-2 = human colon adenocarcinoma cell line, CNS = central nervous system, CYP = cytochrome P, LD_50_ = median lethal dose, and OCT2 = organic cation transporter 2.

**Table 4:** Predicted toxicity endpoints of TOP1 and TOP2 compounds evaluated using the ProTox-II web server. Toxicity parameters include predicted toxicity class, LD_50_, organ-specific toxicity (e.g., hepatotoxicity, cardiotoxicity, nephrotoxicity), and potential interactions with nuclear receptor signaling and stress response pathways.

Endpoint	TOP1	TOP2
Nephrotoxicity	Probably active	Inactive
Cardiotoxicity	Probably active	Inactive
Carcinogenicity	Probably active	Inactive
Aryl hydrocarbon receptor	Inactive	Inactive
Androgen receptor	Inactive	Inactive
Androgen receptor ligand-binding domain	Inactive	Inactive
PPAR-γ	Inactive	Inactive
Nrf2/ARE pathway	Probably active	Inactive
Heat-shock factor response element	Inactive	Inactive
MMP	Inactive	Inactive
p53 pathway	Inactive	Inactive

Abbreviations: MMP = mitochondrial membrane potential, Nrf2/ARE = nuclear factor erythroid 2-related factor 2/antioxidant responsive element, and PPAR-γ = peroxisome proliferator-activated activator gamma.

## Data Availability

The original contributions presented in this study are included in the article/Supplementary Material. Further inquiries can be directed to the corresponding authors.

## Supplementary Materials

The following supporting information can be downloaded at: https://www.mdpi.com/article/10.3390/ijms27135834/s1.

## Abbreviations

The following abbreviations are used in this manuscript:

ADMET	Absorption, distribution, metabolism, excretion and toxicity
ARE	Antioxidant responsive element
ATAD5	ATPase family AAA domain containing 5
BBB	Blood–brain barrier
Caco-2	Human colon adenocarcinoma cell line
CNS	Central nervous system
CYP	Cytochrome P
fraction cSP^3^	Fraction of sp^3^-hybridized carbon atoms
G	Gibbs free energy
GROMACS	GROningen Machine for Chemical Simulation
IsdB	Iron-regulated surface determinant B
LD_50_	Median lethal dose
MDS	Molecular dynamics simulation
MMP	Mitochondrial membrane potential
MMPBSA	Molecular Mechanics Poisson–Boltzmann Surface Area
MRSA	Methicillin-resistant *Staphylococcus aureus*
NEAT	Near-iron Transporter
Nrf2	Nuclear factor erythroid 2-related factor 2
OCT	Organic cation transporter
pkCSM	Small molecule pharmacokinetics prediction
PPAR-γ	Peroxisome proliferator-activated activator gamma
RCSB PDB	Research Collaboratory for Structural Bioinformatics Protein Data Bank
Rg	Radius of gyration
RMSD	Root mean square deviation
RMSF	Root mean square fluctuation
*S. aureus*	*Staphylococcus aureus*
SASA	Solvent-accessible surface area
TOP1	(4-(1-oxoisoindolin-2-yl)benzoic acid)
TOP2	(4-(2-oxochromen-3-yl)benzoic acid)
TPSA	Topological polar surface area

## References

[1] Gherardi G. (2023). *Staphylococcus aureus* Infection: Pathogenesis and antimicrobial resistance. Int. J. Mol. Sci..

[2] Kumar G., Shukla S., Agarwal S., Shukla D., Soni S.R., Verma R.K., Singh V., Kumar A., Kumar D. (2025). Methicillin-resistant *Staphylococcus aureus* in urinary tract infections: A comprehensive review with insights from a north Indian cohort. Cureus.

[3] Talapan D., Sandu A.M., Rafila A. (2023). Antimicrobial resistance of *Staphylococcus aureus* isolated between 2017 and 2022 from infections at a tertiary care hospital in Romania. Antibiotics.

[4] Bassetti M., Righi E., Del Giacomo P., Sartor A., Ansaldi F., Trucchi C., Alicino C., Trecarichi E.M., Spanu T., Paganino C. (2018). Predictors of mortality with *Staphylococcus aureus* bacteremia in elderly adults. J. Am. Geriatr. Soc..

[5] van Hal S.J., Jensen S.O., Vaska V.L., Espedido B.A., Paterson D.L., Gosbell I.B. (2012). Predictors of mortality in *Staphylococcus aureus* bacteremia. Clin. Microbiol. Rev..

[6] Kaushik A., Kest H., Sood M., Thieman C., Steussy B.W., Padomek M., Gupta S. (2024). Infective endocarditis by biofilm-producing methicillin-resistant *Staphylococcus aureus*-pathogenesis, diagnosis, and management. Antibiotics.

[7] Almasri D., Dahman Y. (2023). Prosthetic joint infections: Biofilm formation, management, and the potential of mesoporous bioactive glass as a new treatment option. Pharmaceutics.

[8] Briki A., Alkhatib N., Aloba B., Verma S., Nassar R., Everett D., Ehricht R., Monecke S., Senok A. (2025). Molecular epidemiology of methicillin-resistant *Staphylococcus aureus* in Gulf Cooperation Council countries (2010–2025): A scoping review. Front. Microbiol..

[9] Liu Q., He D., Wang L., Wu Y., Liu X., Yang Y., Chen Z., Dong Z., Luo Y., Song Y. (2024). Efficacy and safety of antibiotics in the treatment of methicillin-resistant *Staphylococcus aureus* (MRSA) infections: A systematic review and network meta-analysis. Antibiotics.

[10] Nandhini P., Kumar P., Mickymaray S., Alothaim A.S., Somasundaram J., Rajan M. (2022). Recent developments in methicillin-resistant *Staphylococcus aureus* (MRSA) treatment: A review. Antibiotics.

[11] Marchetti M., De Bei O., Bettati S., Campanini B., Kovachka S., Gianquinto E., Spyrakis F., Ronda L. (2020). Iron metabolism at the interface between host and pathogen: From nutritional immunity to antibacterial development. Int. J. Mol. Sci..

[12] Murdoch C.C., Skaar E.P. (2022). Nutritional immunity: The battle for nutrient metals at the host-pathogen interface. Nat. Rev. Microbiol..

[13] Ghssein G., Ezzeddine Z. (2022). The key element role of metallophores in the pathogenicity and virulence of *Staphylococcus aureus:* A review. Biology.

[14] Hijazi S., Cozzi M., Asgharpour S., De Bei O., Faggiano S., Marchesani F., Ronda L., Marchetti M., Gianquinto E., Failla M. (2025). First-in-class inhibitors of SbnA reduce siderophore production in *Staphylococcus aureus*. FEBS J..

[15] Prommachote W., Deeudom M., Koonyosying P., Srichomphoo P., Somnabut R., Khamnoi P., Cilibrizzi A., Ravikumar Y., Srichairatanakool S. (2025). Drug susceptibility, siderophore production, and genome analysis of *Staphylococcus aureus* clinical isolates from a university hospital in Chiang Mai, Thailand. Antibiotics.

[16] Ellis-Guardiola K., Mahoney B.J., Clubb R.T. (2020). NEAr transporter (NEAT) domains: Unique surface displayed heme chaperones that enable Gram-positive bacteria to capture heme-iron from hemoglobin. Front. Microbiol..

[17] Hammer N.D., Skaar E.P. (2011). Molecular mechanisms of *Staphylococcus aureus* iron acquisition. Annu. Rev. Microbiol..

[18] Krishna Kumar K., Jacques D.A., Pishchany G., Caradoc-Davies T., Spirig T., Malmirchegini G.R., Langley D.B., Dickson C.F., Mackay J.P., Clubb R.T. (2011). Structural basis for hemoglobin capture by *Staphylococcus aureus* cell-surface protein, IsdH. J. Biol. Chem..

[19] Spirig T., Malmirchegini G.R., Zhang J., Robson S.A., Sjodt M., Liu M., Krishna Kumar K., Dickson C.F., Gell D.A., Lei B. (2013). *Staphylococcus aureus* uses a novel multidomain receptor to break apart human hemoglobin and steal its heme. J. Biol. Chem..

[20] Mishra A.S., Vasanthan M., Malliappan S.P. (2024). Drug repurposing: A leading strategy for new threats and targets. ACS Pharmacol. Transl. Sci..

[21] Ferreira L.G., Dos Santos R.N., Oliva G., Andricopulo A.D. (2015). Molecular docking and structure-based drug design strategies. Molecules.

[22] Lionta E., Spyrou G., Vassilatis D.K., Cournia Z. (2014). Structure-based virtual screening for drug discovery: Principles, applications and recent advances. Curr. Top. Med. Chem..

[23] Medina-Franco J.L. (2021). Computational approaches for the discovery and development of pharmacologically active natural products. Biomolecules.

[24] Clatworthy A.E., Pierson E., Hung D.T. (2007). Targeting virulence: A new paradigm for antimicrobial therapy. Nat. Chem. Biol..

[25] Rasko D.A., Sperandio V. (2010). Anti-virulence strategies to combat bacteria-mediated disease. Nat. Rev. Drug Discov..

[26] Sheldon J.R., Heinrichs D.E. (2015). Recent developments in understanding the iron acquisition strategies of gram positive pathogens. FEMS Microbiol. Rev..

[27] Pishchany G., Skaar E.P. (2012). Taste for blood: Hemoglobin as a nutrient source for pathogens. PLoS Pathog..

[28] Choby J.E., Skaar E.P. (2019). *Staphylococcus aureus* coproporphyrinogen III oxidase is required for aerobic and anaerobic heme synthesis. mSphere.

[29] Bissantz C., Kuhn B., Stahl M. (2010). A medicinal chemist’s guide to molecular interactions. J. Med. Chem..

[30] Warren J.J., Forsberg L.J., Beese L.S. (2006). The structural basis for the mutagenicity of O(6)-methyl-guanine lesions. Proc. Natl. Acad. Sci. USA.

[31] Hollingsworth S.A., Dror R.O. (2018). Molecular dynamics simulation for all. Neuron.

[32] Paradee N., Janthip R., Taesothikul T., Kanjanapothi K., Settakorn K., Srichairatanakool S., Koonyosying P. (2023). Antioxidant and biological activities of Mahajanaka mango pulp extract in murine models. Appl. Sci..

[33] Veber D.F., Johnson S.R., Cheng H.Y., Smith B.R., Ward K.W., Kopple K.D. (2002). Molecular properties that influence the oral bioavailability of drug candidates. J. Med. Chem..

[34] Coordinators N.R. (2017). Database resources of the national center for biotechnology information. Nucleic Acids Res..

[35] Patra S., Paul A., Shand H., Ghosal S., Ghorai S. (2025). In silico identification of anticancer flavonoids as dengue virus replication inhibitors: A molecular docking and simulation approach. J. Mol. Model..

[36] Ghosh S., Chetia D., Gogoi N., Rudrapal M. (2021). Design, molecular docking, drug-likeness, and molecular dynamics studies of 1,2,4-trioxane derivatives as novel *Plasmodium falciparum* falcipain-2 (FP-2) inhibitors. BioTechnologia.

[37] Trott O., Olson A.J. (2010). AutoDock Vina: Improving the speed and accuracy of docking with a new scoring function, efficient optimization, and multithreading. J. Comput. Chem..

[38] Huang J., Rauscher S., Nawrocki G., Ran T., Feig M., de Groot B.L., Grubmuller H., MacKerell A.D. (2017). CHARMM36m: An improved force field for folded and intrinsically disordered proteins. Nat. Methods.

[39] Kumari R., Kumar R., Lynn A., Open Source Drug Discovery Consortium (2014). *g_mmpbsa*—A GROMACS tool for high-throughput MM-PBSA calculations. J. Chem. Inf. Model..

[40] Di L., Kerns E.H. (2003). Profiling drug-like properties in discovery research. Curr. Opin. Chem. Biol..

[41] Banerjee P., Eckert A.O., Schrey A.K., Preissner R. (2018). ProTox-II: A webserver for the prediction of toxicity of chemicals. Nucleic Acids Res..

