# Supplementary material for: In Silico Prioritisation of Similarity-Selected Small Molecules Targeting the IsdB NEAT Domain of Staphylococcus aureus as a Potential Antivirulence Strategy
Source: Int J Mol Sci. 2026 Jun 28;27(13):5834. doi: 10.3390/ijms27135834 (PMC13362405; doi:10.3390/ijms27135834)
Supplement: Supplementary file 1 [file ijms-27-05834-s001.zip › ijms-4319671-supplementary.pdf]

Supplementary Figures

Table S1. Chemical structures and PubChem information of the top-rank compounds (TOP1-TOP10).

Compound	PubChem CID	Chemical name	Molecular formula	Molecular weight (g/mol)
TOP1	13190987	4-(1-Oxoisoindolin-2-yl)benzoic acid	$C_{15}H_{11}NO_3$	253.25
TOP2	675423	4-(2-Oxochromen-3-yl)benzoic acid	$C_{16}H_9O$	265.24
TOP3	53228598	2-Fluoro-4-[2-methoxy-5-(trifluoromethyl)phenyl]benzoic acid	$C_{15}H_{10}F_4O_3$	314.23
TOP4	684763	4-(Cyclopropyliminomethyl)-2-(4-nitrophenyl)-5-(trifluoromethyl)-1H-pyrazol-3-one	$C_{14}H_{11}F_3N_4O_3$	340.26
TOP5	721996	5-Methyl-2-(4-nitrophenyl)-1H-pyrazol-3-one	$C_{10}H_9N_3O_3$	219.2
TOP6	832171	4-(N-Cyclopropyl-C-methylcarbonimidoyl)-5-methyl-2-(4-nitrophenyl)-1H-pyrazol-3-one	$C_{15}H_{16}N_4O_3$	300.31
TOP7	872756	2-(4-Nitrophenyl)-5-propyl-1H-pyrazol-3-one	$C_{12}H_{13}N_3O_3$	247.25
TOP8	889081	4-(3-Oxo-5-phenyl-1H-pyrazol-2-yl)benzoic acid	$C_{16}H_{12}N_2O_3$	280.28
TOP9	890848	2-(4-Nitrophenyl)-5-(trifluoromethyl)-1H-pyrazol-3-one	$C_{10}H_6F_3N_3O_3$	273.17
TOP10	976591	4-(3-Oxo-5-propyl-1H-pyrazol-2-yl)benzoic acid	$C_{13}H_{14}N_2O_3$	246.26

A B C

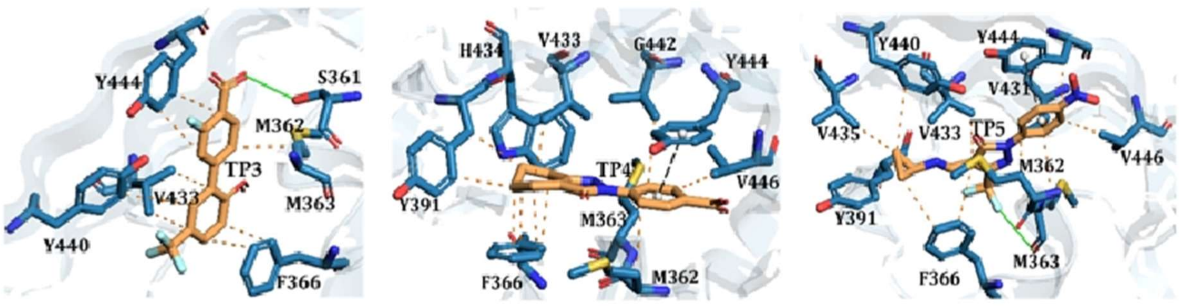

D

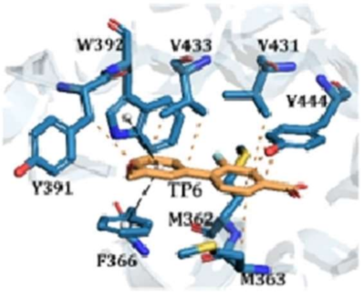

E

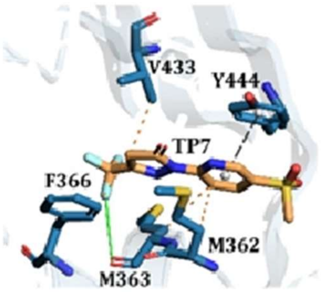

F

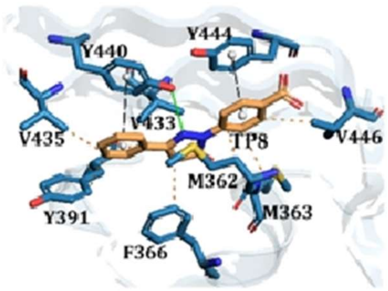

G

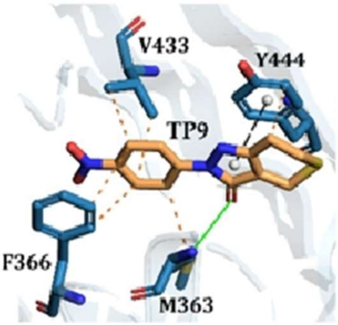

H

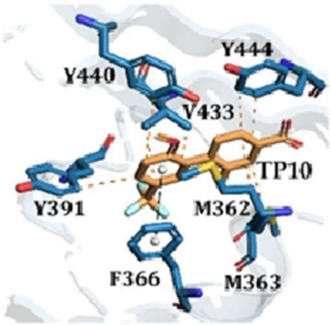

Figure S1. Three-dimensional binding poses of similarity-selected small molecules docked within the IsdB NEAT domain (PDB ID: 3RTL). (A–H) Stick representations of the top-ranked compounds (TOP3–TOP10) within the heme-binding pocket of the IsdB NEAT domain, illustrating ligand orientation and spatial accommodation in the binding site.

A

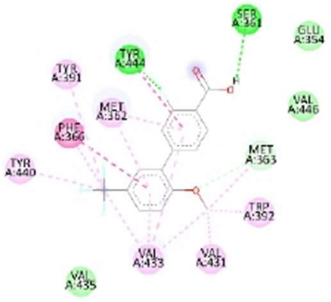

B

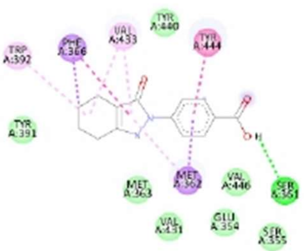

C

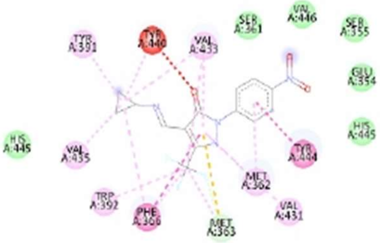

D

E

F

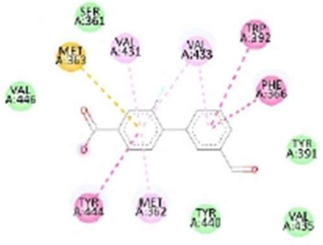

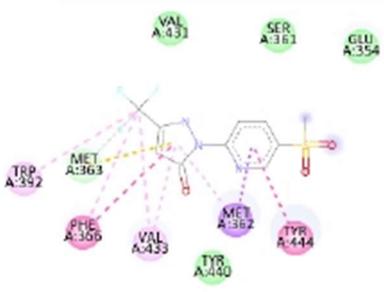

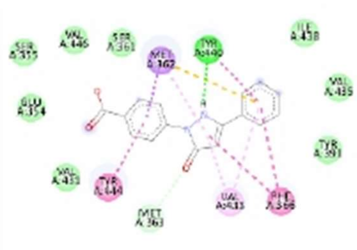

G

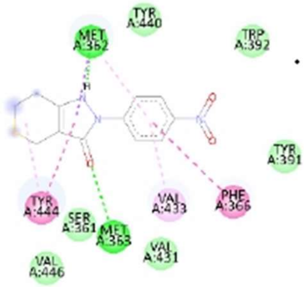

H

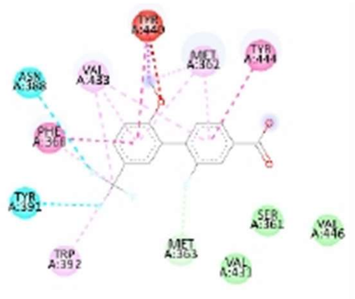

Figure S2. Two-dimensional interaction diagrams of similarity-selected small molecules docked within the IsdB NEAT domain (PDB ID: 3RTL). (A–H) Stick representation of selected compounds (TOP3–TOP10), highlighting hydrogen bonds, hydrophobic contacts, and other non-covalent interactions with key residues in the heme-binding pocket.
